# Commentary: Ethological Evaluation of the Effects of Social Defeat Stress in Mice: Beyond the Social Interaction Ratio

**DOI:** 10.3389/fnbeh.2016.00155

**Published:** 2016-08-10

**Authors:** Darya A. Meshalkina, Allan V. Kalueff

**Affiliations:** Institute of Translational Biomedicine, St. Petersburg State UniversitySt. Petersburg, Russia

**Keywords:** social stress, chronic social defeat stress, mice

The social defeat paradigm, originally developed by N. Kudryavtseva's group in early 1990s (Kudryavtseva and Bakshtanovskaya, 1989; Kudryavtseva et al., 1991; Kudryavtseva, 1994), has become a widely used model of chronic social stress (Berton et al., 2006; Krishnan et al., 2007; Golden et al., 2011; Veerakumar et al., 2014; Smagin et al., 2016). A recent study (Henriques-Alves and Queiroz, 2015) has critically evaluated traditional and novel behavioral endpoints in this model, calling for assessing multiple behaviors (rather than single indices) in longer test sessions to correctly interpret behavioral data. In line with the renowned psychopharmacologist Prof. Slava Lapin (“one experiment is not an experiment; one behavior is not a model”), these results also corroborate our long-held argument that reduced activity in the stimulus zone of the social defeat model may not necessarily reflect rodent depression-like state (e.g., social withdrawal), but can also be relevant to other traits, especially anxiety (Kudryavtseva et al., 1991; Kudryavtseva, 2003; Kalueff et al., 2006). It is appreciated that similar conclusions have been reached in a recent thorough study of mouse social defeat behaviors (Henriques-Alves and Queiroz, 2015).

However, several additional considerations may factor into their experimental findings and data interpretation, thereby meriting further in-depth discussion here. For example, using a 5-day social defeat stress protocol, the authors suggest that anxiety- and depression-related behaviors may reflect the mouse “difficulty … in evaluating reward and threat respectively,” p. 2 (Henriques-Alves and Queiroz, 2015). However, in our own experience with this model, 5 days of repeated confrontations may be merely insufficient to develop depression-like phenotype in mice, where much longer (typically, 20-day) periods of daily fighting are needed to evoke such states (Kudryavtseva et al., 1991; Kudryavtseva, 2000, 2003, 2009; Avgustinovich et al., 2005; Kalueff et al., 2006). In contrast, anxiety-like responses can be the likely phenotype evoked by shorter manipulations in the social defeat paradigm. For instance, 10 days of chronic social defeat stress evoke mouse anxiety-like behaviors which were corrected by anxiolytic drugs but not antidepressants (Kudryavtseva et al., 1991; Kudryavtseva, 2000, 2003; Avgustinovich et al., 2005; Kalueff et al., 2006; Kudryavtseva, 2009). Thus, it is likely that anxiety-like states (which also often manifest as reduced social investigation), rather than depression-like pathogenesis, could have been evoked in Henriques-Alves and Queiroz (2015) utilizing a similarly short 5-day repeated social stress protocol.

Importantly, the social defeat stress protocol itself is subject to a substantial variance across the laboratories worldwide (Kudryavtseva, 2009; Golden et al., 2011), and these modifications represent as a healthy discourse of scientific inquiry. In particular, the discussed study used a popular C57BL/6J mouse strain confronted with heavier, sexually-experienced, socially isolated and thereby more aggressive resident Swiss mice (Henriques-Alves and Queiroz, 2015). On one hand, this experimental design, albeit sufficient for the purpose of evoking social defeat *per se*, raises the question of whether the social stress in this context was “fierce” enough for the C57BL/6J mice (as compared, for example, with a situation which can be modeled by confronting two same-size mice of the same strain, e.g., C57BL/6J). The strain selection is an interesting and yet under-investigated problem in the social stress paradigms which merits some consideration. One hypothetical possibility, for example, is that the same-strain fighting might be more intense because more fights and/or more severe fighting can be needed to determine the winner among the two similar (vs. phenotypically different) members of the fighting pairs. Indeed, while avoiding to anthropomorphize animal models, it is hard to argue that a boxing match is more intense and lasts longer when two boxers are of similar weight and strength. Respectively, this possibility may translate into a stronger social stress in the former (vs. the latter) cases, and the same logic can apply to mouse strains in the social stress paradigms. On the other hand, the use of an unfamiliar Swiss mouse as a social stimulus in the second “social” session of the social interaction test in Henriques-Alves and Queiroz (2015) raises methodological questions of whether there was, in fact, a genuine *social* avoidance in defeated mice in this experimental design, or a *victim-like* avoidance of stronger “predator” mice (i.e., based on conditioned generalization for stressed C57BL/6J mice to avoid any Swiss mice, perceived as a priori more aggressive and stronger). Robust differences in mouse strains' physical appearance (size, fur color) may make such conditioned avoidance for the tested C57BL/6J mice easier to develop. For example, given the potentially severe nature of mouse aggressive confrontations, such avoidance in defeated C57BL/6J mice could have had a conditioned nature, reflecting a post-traumatic stress disorder (PTSD)-like response to a sight of a stronger, heavier “winner” stimulus strain (e.g., similar to PTSD-like learned avoidance of rodents of a predator snake, see Figure 1). Although the potential of rodent social stress studies to model PTSD pathogenesis remains to be tested, the poly-strain study designs similar to Henriques-Alves and Queiroz (2015) may represent a promising strategy for this line of research. From this point of view, the selection of C57BL/6J or another (e.g., “neutral,” non-Swiss) mouse strain as a social stimulus could therefore provide an additional critical dissection of various experimental factors contributing to the resultant observed phenotype.

**Figure 1:**
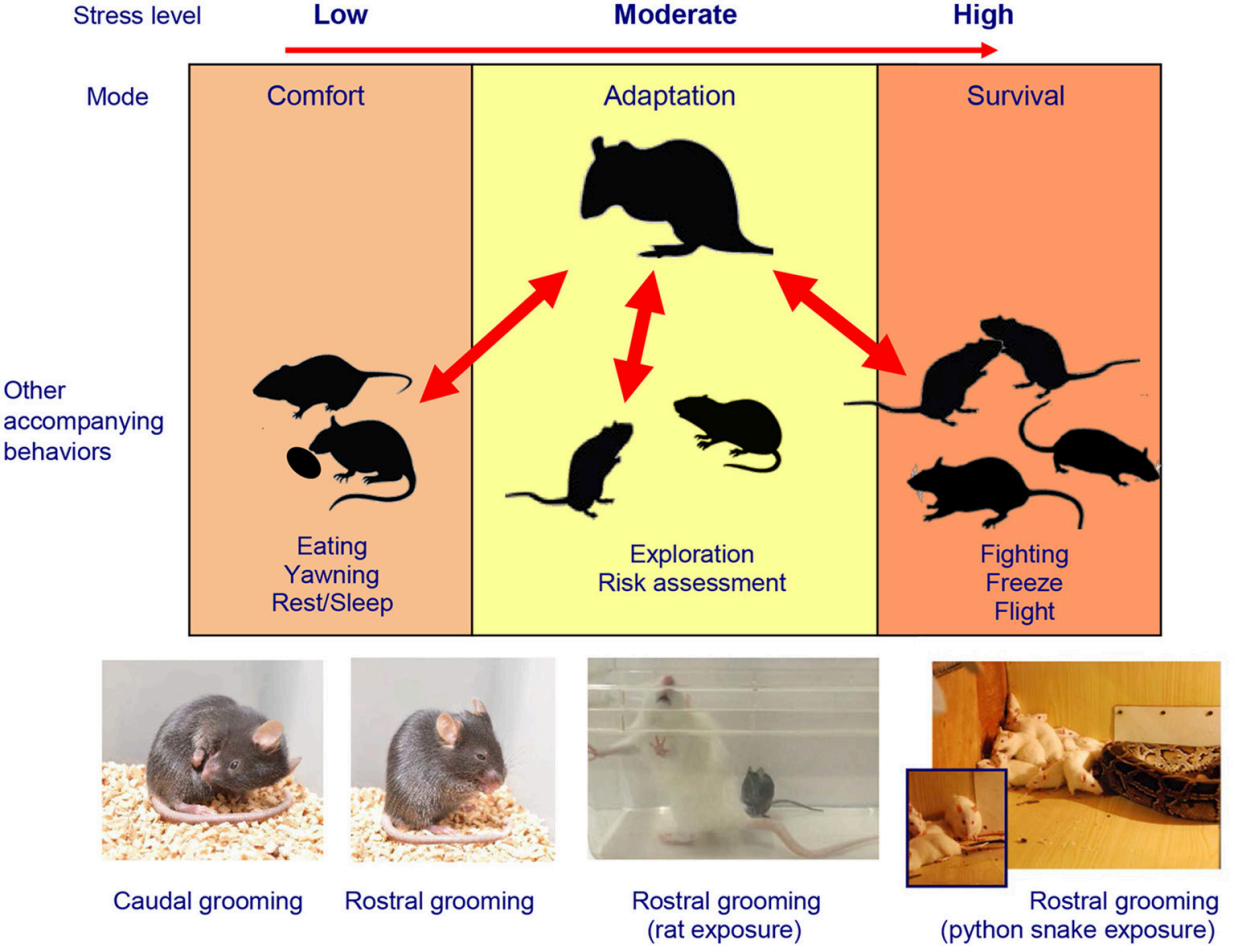
**Stress-related modulation of rodent self-grooming phenotypes**. The complex multi-factorial nature of rodent self-grooming includes “comfort” self-grooming in low-arousal, elevated self-grooming in moderate arousal conditions and “fight, flight, freeze, or groom” stress response (Kalueff et al., 2016; Song et al., 2016) in high-arousal context, highly relevant to the social defeat model (Kudryavtseva and Bakshtanovskaya, 1989; Kudryavtseva, 2000, 2009). Also note predominantly less frequent but longer, caudal (body/tail/genitals) self-grooming typical for low-arousal “comfort” states, which differs from mostly short, rostral (paws/face/head) grooming seen in high-stress situations. Photos illustrate examples of stress-evoked self-grooming in several rodent models, including caudal and rostral self-grooming observed in C57BL/6J mice in relatively moderate aversive conditions (e.g., bright light and novelty exposure) and overt extra-short bouts of rostral self-grooming during severe stressors. Note frequent rostral self-grooming occurring in these mice during the predator (rat) exposure test, especially prior or after attach or follow by a rat. Frequent rostral self-grooming also occurs in rodents during the predator exposure (e.g., in rats exposed to a large python snake), especially after witnessing the snake attack on a peer Wistar rat (images—courtesy of Drs. N. Kudryavtseva, D. Smagin, V. Klimenko, and S. Tsikunov).

Another interesting observation made in this study concerns habituation of various behavioral endpoints over time, since only control mice showed habituation of social investigation of the unfamiliar conspecific (Henriques-Alves and Queiroz, 2015). However, this finding can be unsurprising, considering the complex interplay between memory and anxiety in rodent stress-based behavioral models, and the well-established ability of chronic stress to impair cognitive functions in both humans and animals; see Kalueff and Nutt (1996) and Kalueff and Murphy (2007) for details. At first glance, failure to habituate observed only for social investigation, but not for novel object investigation (Henriques-Alves and Queiroz, 2015), downplays the role of memory-anxiety interaction. Nevertheless, these data do not necessarily negate the impact of cognitions on the observed mouse phenotypes. For example, if anxiety-memory interplay more selectively affects social anxiety than other (e.g., more generalized) subtypes of anxiety, then habituation of social investigation can suffer more than that of non-social investigation. In fact, it is rather logical to expect that prior social stress experience may affect social investigation more strongly than the other types of anxiety-like behaviors, such as novel object investigation.

Yet another potential explanation for this phenotype may be that stressed “conditioned” defeated C57BL/6J mice could develop an adaptive high-vigilance behavior which they, unlike normally habituating controls, maintain at a constant level, as part of their “protective” behavioral strategies. In the latter case, for example, the very adaptive nature of vigilance behavior presumes the constant level of protective alertness, and is often seen in various species, from fish to rodents (Stewart et al., 2012), as non-habituated behaviors.

Overall, Henriques-Alves and Queiroz performed an excellent ethological analysis of their mouse cohorts, timely focusing on stratification of defeated mice into sub-groups based on their behavioral performance in various tests (Henriques-Alves and Queiroz, 2015). Notably, however, sub-groups of “resilient” and “susceptible” animals selected based on their social avoidance index did not differ in sucrose preference (Henriques-Alves and Queiroz, 2015), further supporting the possibility that mouse depression-like phenotype may, in fact, not be evoked by the utilized experimental design (note, however, that studies using longer chronic stress, e.g., Berton et al., 2006; Krishnan et al., 2007 did show reduced sucrose preference in the socially defeated mouse cohorts). Furthermore, the stratification of mouse cohorts into resilient and susceptible, as logically note the originators of the chronic stress paradigm (Kudryavtseva, 2009), may or may not be due to pre-determined superiority of one individual over another in their coping strategies. For example, why would highly inbred and uniformly housed mice react in a different way to a similar intimidating impact? (Kudryavtseva, 2009). Indeed, an alternative explanation for this divergence can be learned experience of social defeat per se, determined by methodological factors—e.g., by the outcome of mouse first fighting experiences (Kudryavtseva, 2009). For example, discussing an example similar to the small/large mouse experimental design used by Henriques-Alves and Queiroz (2015), and based on extensive first-hand experience with chronic social defeat model, Kudryavtseva notes that during the first confrontations, the repeatedly defeated (loser) mice can be learning adaptive behavioral strategies that dampen strong aggression in a larger male and, thus, adjust their coping accordingly, anticipating lower stress from a winner mouse (Kudryavtseva, 2009). Of note, while various laboratories may use different criteria for selecting loser and winner mice in the chronic social stress model (Kudryavtseva, 2009; Golden et al., 2011), it is usually done by assessing animal agonistic behaviors, such as sniffing, touching, dominant hetero-grooming, chasing and biting (Denmark et al., 2010). After each social confrontation, in addition to a clear dominant winner mouse, a passive “loser” mouse would typically emerge, usually displaying defensive behaviors, such as sideways or upright submissive postures, withdrawal, fleeing, lying on its back or freezing (Denmark et al., 2010). As assessed by daily “win-or-lose” scoring, winners can be defined as mice dominant in ≥70–80%, and losers as animals experiencing only ≤ 20% of victories, during the entire duration of the social defeat paradigm (Denmark et al., 2010).

Finally, whereas detailed ethological analyses were performed in this study, other relevant stress-related behaviors, such as freezing and self-grooming, were not assessed (Henriques-Alves and Queiroz, 2015). Importantly, since these additional behaviors may be an essential part of the rodent “stress” (fight, flight, freeze or groom) response (Song et al., 2016) (Figure 1), they may be predictably activated in “stressed” defeated mice following their novelty-based social interaction testing 24 h after the last defeat. Thus, increased grooming or freezing activity, if observed as part of baseline anxious phenotype of socially stressed mice, may confound the overall expression of their social and locomotor behaviors, thereby affecting the social interaction ratio because various stress-related behaviors would compete with each other for the test time and motor actions (e.g., animals cannot groom and socially investigate at the same time). Moreover, all these stress-induced behaviors may represent *learned* (e.g., PTSD-like) responses to a social interaction with a specific perceived threat—the Swiss mouse presentation. In this case, the aversion of a Swiss mouse (established during repeated defeats) may translate into reduced social exploration during the social interaction trial, when the same “threatening” strain is presented 24 h later. In turn, such re-experience stress may further elevate non-exploratory anxiety-like behaviors, including grooming and freezing (which, as noted above, can even more confound social investigation indices).

Interestingly, following 15–17 days of chronic social defeat stress, defeated male C57BL/6J mice exhibited a disorganized patterning (sequencing, Figure 1) of their self-grooming behaviors, which emerged as a behavioral marker of chronic social stress, both immediately after social confrontation and 24 h later (Denmark et al., 2010). Together, these findings suggest that chronic social stress modulates self-grooming behavior in mice, supporting the emerging importance of self-grooming phenotypes in the social defeat model (Denmark et al., 2010). However, it is unclear to which extent such non-social/non-avoidance behaviors may share common “high-stress” circuits with social investigation, or compete with stress-evoked social responses for neural circuitry and motor movements (Kalueff et al., 2016; Song et al., 2016). The value of such behaviors (beyond the social interaction domain) for modeling human and animal affective conditions have been already recognized (Kalueff et al., 2007; Stewart and Kalueff, 2015), further helping to disentangle anxiety- and depression-like phenotypes in the social defeat paradigm. Again, as already mentioned, pharmacological validation of the observed phenotypes by using anxiolytic and antidepressant drugs may help further dissect between anxiety- and depression-related behaviors in mouse chronic social stress models.

Accordingly, only focusing on a wider spectrum of mouse social and non-social behaviors, and moving away from over-reliance on single “interaction” endpoints, will foster a more detailed ethological evaluation of the effects of social defeat stress in mice. In addition to this, we need a better understanding of environmental/epigenetic landscape as well as cellular/molecular mechanisms and neural circuits that contribute to animal social stress responses, and how they translate into human affective brain disorders.

## Author contributions

All authors listed have made substantial, direct and intellectual contribution to the work, and approved it for publication.

### Conflict of interest statement

The authors declare that the research was conducted in the absence of any commercial or financial relationships that could be construed as a potential conflict of interest.
